# T Cell Receptor Chain Centricity: The Phenomenon and Potential Applications in Cancer Immunotherapy

**DOI:** 10.3390/ijms242015211

**Published:** 2023-10-16

**Authors:** Anastasiia A. Kalinina, Ludmila M. Khromykh, Dmitry B. Kazansky

**Affiliations:** N.N. Blokhin National Medical Research Center of Oncology of the Ministry of Health of the Russian Federation, 115478 Moscow, Russia

**Keywords:** cancer immunotherapy, adoptive cell therapy, T cell receptor, chain centricity, dominant-active hemi-chain TCR

## Abstract

T cells are crucial players in adaptive anti-cancer immunity. The gene modification of T cells with tumor antigen-specific T cell receptors (TCRs) was a milestone in personalized cancer immunotherapy. TCR is a heterodimer (either α/β or γ/δ) able to recognize a peptide antigen in a complex with self-MHC molecules. Although traditional concepts assume that an α- and β-chain contribute equally to antigen recognition, mounting data reveal that certain receptors possess chain centricity, i.e., one hemi-chain TCR dominates antigen recognition and dictates its specificity. Chain-centric TCRs are currently poorly understood in terms of their origin and the functional T cell subsets that express them. In addition, the ratio of α- and β-chain-centric TCRs, as well as the exact proportion of chain-centric TCRs in the native repertoire, is generally still unknown today. In this review, we provide a retrospective analysis of studies that evidence chain-centric TCRs, propose patterns of their generation, and discuss the potential applications of such receptors in T cell gene modification for adoptive cancer immunotherapy.

## 1. Introduction

T lymphocytes are one of the major effectors of adaptive immunity, including anti-cancer immunity. To achieve their functions, these cells are equipped with a highly specialized receptor, the T cell receptor (TCR), which recognizes a peptide antigen in a complex with self-MHC molecules (pMHC complex). TCR is a heterodimer consisting of either α- and β-chains or γ- and δ-chains. This review is focused on the predominant form, TCRαβ.

Adoptive cell therapy (ACT) is a powerful strategy in the treatment of cancer and infectious diseases [1,2,3,4,5,6]. The gene modification of patients’ T lymphocytes with tumor antigen-specific TCRs or chimeric antigen receptors (CARs) has become a milestone in the immunotherapy of hematological and solid malignancies. Although CAR-T cells are advantageous due to their capability of recognizing target antigens in a non-MHC-restricted manner, such T cells cannot be engineered to recognize tumor neoantigens. In contrast, TCR-T cells can target both tumor-associated and tumor-specific antigens in complex with self-MHC molecules. Therefore, TCR-T cell ACT is more versatile and can be applied to more cancer types. Yet, because of HLA polymorphisms, TCR-T cell ACT is always personalized.

The classical paradigm states the equal contribution of both hemi-chains TCR to antigen recognition. The mechanisms of TCR binding to a pMHC complex have been thoroughly reviewed elsewhere [7,8]. Yet, cumulating data suggest it is not the case for some TCRs. Years ago, both in experimental animals and humans, the asymmetric roles of either hemi-chain TCR in antigen recognition were acknowledged. But it was only in 2015 that the group of Dr. Naoto Hirano from the Princess Margaret Cancer Centre (Toronto, Ontario, Canada) introduced the special term “TCR chain centricity” to describe this phenomenon and define a T cell receptor, in which either hemi-chain dominates in antigen recognition and dictates its specificity [9,10]. Such TCRs were, therefore, called chain-centric.

Although the existence of chain-centric TCRs is currently undeniable, there are still many problems with this phenomenon. The first fundamental question concerns the mechanisms of such receptors’ formation. As no direct experimental data are available to date, one can speculate that their generation could be stochastic during thymic T cell development or guided by the rules of TCR editing or revision. This raises the second question: what functional T cell subsets (i.e., antigen-unchallenged T cells, effectors, or memory T cells) can express chain-centric TCRs? These issues make the identification and isolation of T lymphocytes with such receptors challenging. Therefore, little is known about the actual proportion of chain-centric receptors in the native TCR repertoire. The true ratio of α- and β-chain-centric TCRs has also yet to be determined. Despite these fundamental issues, however, we assume that the practical application of such receptors in cancer T cell adoptive therapy could be beneficial, with the potential to improve its safety and applicability while reducing expenses.

In this review, we discuss the phenomenon of TCR chain centricity and analyze studies that evidence such receptors. We also posit theories on the patterns of their origin and suggest potential applications of chain-centric receptors in T cell gene editing techniques for adoptive cancer immunotherapy. But first, it might be helpful to briefly describe the generation of TCR during T cell development.

## 2. Formation of a T Cell Receptor during T Cell Development

T cell progenitor commitment and maturation occur in the thymus and are critically dependent on Notch1, a transmembrane receptor expressed on thymic epithelial cells [11]. Notch1 dimerization activates the expression of recombination activating genes (RAG) 1 and 2, which encode site-specific endonucleases that create double-stranded breaks in the process of V(D)J gene recombination during α/β-chain TCR assembly [12]. 

In an immature thymocyte, TCR shaping starts with the gene rearrangement of a β-chain from one allele. Productive in-frame VDJ rearrangement generates a β-chain capable of pairing with the surrogate α-chain to form a pre-TCR [13]. The assembly of the pre-TCR/CD3 complex provides constitutive signaling that induces thymocyte proliferation and inhibits RAG-1 and RAG-2 expression, blocking further TCRβ gene rearrangement on the second locus [14]. Thus, TCRβ rearrangement is guided by the rule of allelic exclusion.

In double-positive CD4+CD8+ thymocytes, RAG expression is re-induced to initiate TCRα rearrangement [14]. Interestingly, the TCRα gene locus (consisting of V, J, and constant (C) segments) is very expanded and organized in a manner allowing multiple rearrangements on one chromosome [15]. Vα-Jα gene rearrangement must generate a functional α-chain capable of pairing with TCRβ to form a selectable TCR [16,17]. TCRα rearrangement is not restricted by allelic exclusion, and multiple VαJα rearrangements can occur on one locus or simultaneously from both alleles [18,19,20]. 

Both α- and β-chain TCR contain three complementarity-determining regions (CDR), mainly contributing to peptide-MHC recognition. CDR1 and CDR2 are encoded by the V segment, and their diversity is limited by the germline gene segments available for recombination [21,22]. In contrast, the CDR3 region is highly variable, resulting from the random combination of V(D)J segments and the addition of nucleotides in a non-template manner at the V(D)J joints (the N region) [22]. This largely determines the diversity of the TCRαβ repertoire, which is predicted to be 10^14^ in humans [22]. The CDR1 and CDR2 regions primarily make contacts with an MHC molecule, while CDR3 mediates TCR interaction with a docked antigen [8]. Therefore, CDR3α and CDR3β are the structural basis of the clonal specificity of T lymphocytes.

## 3. How the Phenomenon of TCR Chain Centricity Was Discovered?

The asymmetric contribution of either hemi-chain TCR to antigen recognition was acknowledged decades ago, both in humans and experimental animals. This section summarizes results from multiple experimental systems on chain-centric TCRs (Table 1).

### 3.1. Analysis of the Immune TCR Repertoire

In the late 1980s, the first study to provide conclusive proof that a single hemi-chain TCR predominates in antigen recognition was published [23]. Tan et al. studied the immune response to p-azobenzenearsonate in mice and found that transfer of the α-chain of the arsonate-specific TCR with unrelated TCRβ to recipient T cells conferred responsiveness to this hapten [23]. Later, Deckhut et al. analyzed the immune response to influenza A virus in mice to show that CD8+ Vβ8.3+ T cells were predominantly involved in the inflammatory process during the infection and TCRβ played a dominant role in epitope recognition [24].

To further explore the impact of a single α/β-chain in antigen recognition, Yokosuka et al. developed a novel transfection model [25]. The authors used the α-chain derived from TCR of either the RT-1 T cell clone specific to HIV envelope glycoprotein gp160 or the P14 clone specific to lymphocytic choriomeningitis virus (LCMV). TG40 hybridoma lacking its TCRαβ was co-transfected with one of these two α-chains and various β-chains endogenously rearranged in non-immunized wild-type mice. The studies showed that one-third of randomly chosen β-chains could pair with RT-1-TCRα to form a receptor capable of recognizing the cognate antigen. These results suggested the dominant role of the α-chain TCR of the RT-1 clone in antigen recognition, with minimal reliance on a β-chain that contributed only to interaction with the MHC-peptide complex. Although the α-chain TCR of the P14 clone also exhibited a predominant role, its pairing with only 4% of random TCRβ generated a functional antigen-specific receptor, indicating a stronger dependency of the P14-TCR on CDR3β in antigen recognition [25]. 

Nakatsugawa et al. established the human T cell clone with the α-chain-centric TCR specific for the peptide derived from the melanocyte differentiation antigen MART-1 (melanoma antigen recognized by T cells 1) [10]. The transduction of this TCRα into peripheral blood T cells provided reactivity for the specific peptide–HLA complex regardless of the HLA haplotype of T cell donors. Further studies showed that although the dominant α-chain dictated antigen specificity, the paired β-chain changed the receptor avidity without influencing its specificity [10]. 

In 2015, the group led by N. Hirano published another work. The authors analyzed the TAK1 clone specific to the peptide derived from the Wilms tumor antigen in a complex with the HLA-A*24:02 allele and cross-reactive to HLA-B*57:01 [9]. The TCR of this clone possessed the dominant-active β-chain, which dictated both the specific reactivity and cross-reactivity. Interestingly, its pairing with various α-chains reconstituted receptors with enhanced, weakened, or absent specific and/or cross-reactivity [9]. 

In our work, we employed *Salmonella typhimurium* as a model infection in mice to show that chain-centric TCRs are ordinarily generated during the immune response, and T cell clones with the dominant-active antigen-specific α-chain comprise a significant fraction (~20%) of the naturally forming pool of memory T cells [5,6]. We proved that antigen-unchallenged T cells transduced with such dominant-active salmonella-specific TCRα could recognize bacterial antigens both in vitro and in vivo [5]. Furthermore, the adoptive transfer of unprimed T cells transduced with the dominant-active α-chain improved host defense against salmonellosis [5].

### 3.2. Studies Using Single TCRα/β Transgenic Mice

Brändle et al. used two transgenic mouse lines independently expressing the α- or β-chain TCR of the LCMV-specific P14 clone to show that upon cognate infection, specific cytotoxic T lymphocytes (CTLs) in TCRβ-transgenic mice predominantly used highly conserved TCRα [26]. Subsequently, the authors demonstrated that transgenic mice expressing P14-TCRα developed a primary anti-LCMV immune response in vitro in contrast to wild-type animals that required priming in vivo [27]. These results were confirmed and complemented by the later work of Yokosuka et al. (Section 3.1) [25]. 

In their study, Dillon et al. analyzed transgenic mice expressing Vβ5.2 TCRβ derived from the CD8+ cytotoxic clone specific to chicken ovalbumin (OVA) [32]. Vβ5.2+ T cells isolated from non-immunized transgenic mice expressed variable α-chains and exhibited a strong primary anti-OVA response, indicating that the specific immune response was not solely attributed to restoration of the original TCR [32].

Mori et al. exploited the mouse model of collagen-induced arthritis (CIA) and generated transgenic mice expressing the β-chain TCR of the anti-collagen type II-specific arthritogenic T cell clone. This TCRβ contributed to susceptibility to CIA, but its transgenic expression in T cells was insufficient to break resistance in tolerant mice [33].

In contrast to these studies, in transgenic mice carrying the β-chain TCR of the RT-1 clone (specific for HIV gp160), cytotoxic T cells expressed homogeneous TCRα and exhibited a weak but significant immune response to the specific antigen [25]. CTLs in RT-1-TCRα-transgenic mice used random β-chains but still showed specific cytotoxicity comparable to those in transgenic animals expressing the full receptor of the RT-1 clone. These findings confirmed the predominant role of the α-chain of the RT-1-TCR in cognate antigen recognition in vivo and were in compliance with the results of the in vitro system [25] (Section 3.1).

According to several studies, a single hemi-chain of some public TCRs may predominate in the immune response to various antigens [28,40,41]. For example, Zhao et al. investigated the role of individual β-chains of public and private disease-associated TCRs in the development of experimental autoimmune encephalomyelitis (EAE) in mice [28]. The authors found that the transgenic expression of only public β-chains conferred autoantigen reactivity on unprimed T cells. Furthermore, several public β-chains, but not any private β-chains, induced spontaneous early-onset EAE in transgenic mice [28]. 

Recently, we generated transgenic mice that expressed an α-chain TCR of the mouse memory T cell clone 1D1 specific to the H-2K^b^ alloantigen [4]. These transgenic animals exhibited a strong primary immune response to the specific antigen expressed on mouse lymphoma EL-4 cells. Our data showed that TCRα-transgenic mice had an inborn, pre-formed pool of CD8+ effector and memory anti-tumor T cells due to the expression of the single antigen-specific dominant-active TCRα [4]. It is noteworthy that transgenic mice expressing the β-chain TCR of the 1D1 clone were immunodeficient and failed to establish an effective immune response to the specific alloantigen because of the narrowing of TCR repertoire diversity [42,43].

Several studies reported impaired thymic development because transgenic α-chain expression was forced non-physiologically early in double-negative thymocytes [27,44]. Still, the fitting expression of a single transgenic α/β-chain TCR in proper thymocyte subsets [45] was shown not to interfere with T cell development in our experimental models [4,42]. Therefore, we propose that the transgenesis of the dominant-active α-chain could produce animals with improved immunity and no significant alterations in the native TCR repertoire [4,46], as the rearrangements of endogenous α-chains are not permitted by allelic exclusion, and TCRα transgenesis could broaden the TCR repertoire diversity and likely enhance host immune defense [4]. 

## 4. Could α-Chain-Centric TCRs Develop More Frequently?

Although studies have evidenced that either hemi-chain TCR can predominate in antigen recognition (Table 1), the ratio of α-centric and β-centric TCRs, as well as the frequency of their generation, are still unknown. Still, considering certain physiological and functional features of an α-chain, we assume that chain-centric TCRs with a dominant-active α-chain could likely prevail. 

### 4.1. Features of TCRα Generation in a Developing Thymocyte Could Suggest Its Functional Pre-Dominance

The first evidence may lie in the features of TCRαβ generation in a developing thymocyte. As mentioned above, TCRβ gene rearrangement must be productive and generate a β-chain capable of forming a pre-TCR by pairing with a surrogate α-chain [15]. In contrast, a newly generated α-chain must not only be capable of pairing with the rearranged β-chain but also form a receptor with which a thymocyte will pass positive selection [18,20] or even survive negative selection [16,17,19]. To meet these requirements, Vα-Jα genes undergo multiple rearrangements from one allele or simultaneously from both loci [20,47], and α-chains are selected in a process known as TCRα gene editing [16,18,20]. Obviously, newly formed α-chains are subjected to intense selection based on their ability to ensure the interaction of TCR with peptide-MHC complexes [19,47]. Thus, the generation of α-chain-centric TCRs could increase the efficiency of positive selection within the thymus. The process of TCR editing, as one of the proposed mechanisms of chain-centric TCR formation, will be discussed in more detail later (Section 5.1). 

### 4.2. TCRα Can Dictate the Mode of TCR Interaction with pMHC

As mentioned in Section 2, three complementarity-determining regions (CDR) of both α- and β-chain TCR contribute to peptide-MHC binding and recognition. The overall orientation of TCR on a pMHC complex and features of contacts of the CDR3 region(s) with the bound antigen can influence the affinity of receptor–ligand interaction and TCR specificity [7,8,21,34]. 

Stadinski et al. studied receptors, which consisted of one fixed TCRβ paired with variable α-chains [21]. Surface plasmon resonance and the TCR multimer staining assay showed that differential TCRα/β pairing changed the binding affinity of the resultant receptor with the specific pMHC. Furthermore, the CDR3 region of some α-chains had more contacts with the bound peptide. X-ray crystallographic analysis in this study revealed that TCRα could alter conformations of CDR loops of the β-chain and dictate its orientation on a pMHC complex [21].

These findings perfectly complement previous investigations. The mouse T cell clone 2C recognizes the peptide EQYKFYSV (dEV8) in complex with the MHC class I molecule H-2K^b^ [48]. The crystal structure of 2C-TCR bound to this pMHC showed that CDR3β had minimal contacts with the peptide, but TCRα mediated extensive interaction of the receptor with α-helices of the MHC molecule, dictating the orientation of the whole trimolecular complex [29]. Reinherz et al. discovered that the Vα of the MHC class II-restricted TCR dominated in peptide recognition, providing 23 of 27 atomic contacts with it and binding to the β1-helix of the MHC class II molecule [30].

Studies of the public TCR specific to the epitope of Epstein-Barr virus demonstrated that after the antigen encounter, the conformation of the CDR1 and CDR2 loops of its α-chain changed significantly to enhance the interaction with the pMHC [35]. The α-chain with the TCRAV12-2 gene segment was found to be predominant in human T cell clones specific to the peptide derived from MART-1 and presented by the HLA-A2 allele [36,37]. It was shown that its CDR1α and CDR2α loops guided the TCR binding onto the peptide-HLA complex without a significant contribution of heterogeneous β-chains [36,37].

In contrast, several studies indicated that TCRβ could dominate in interactions with pMHC [28,31,34,49]. For example, in the mouse model of myelin oligodendrocyte glycoprotein (MOG)-induced experimental autoimmune encephalomyelitis, MOG-reactive T cell clones shared public TCRβ1 that dominated in contacts with pMHC and conferred the receptor with high functional avidity for the antigen. At the same time, heterogeneous α-chains were only required to maintain interaction integrity and provide additional association energy for effective TCR stimulation [28]. 

Previous findings indicated that TCR contains germline-encoded contact points that provide generic MHC recognition and control thymic selection [50]. In TCRβ-transgenic mice, mutations in CDR2β significantly reduced the diversity of the TCR repertoire [50]. However, several studies pointed out the crucial role of the α-chain elements (the N and J regions, the CDR1 and CDR2 loops) in discriminating between MHC class I- and II-restricted recognition [51,52,53,54]. 

In conclusion, a number of studies have shown that an α-chain can make more contacts with a peptide-MHC complex and alter the way that TCR interacts with it, thereby establishing a structural basis for TCRα dominance. Considering the developmental and functional features of an α-chain, we surmise that α-chain-centric TCRs can form more frequently. The findings of Trautmann et al. could support this hypothesis to some extent [38]. The authors isolated from different donors CTLs that were selected in vivo under acute or chronic exposure to either viral antigens (Epstein-Barr virus or human cytomegalovirus) or melanoma antigens. Surprisingly, all these CTLs had dominant Vα but never Vβ, suggesting that α-chain TCR predominance could be a general event [38]. 

In light of this supposition, our recent bioinformatics study of TCRα/β physicochemical features in tumor antigen-specific memory T cells is of particular interest [55]. We exploited an experimental mouse tumor model to generate primary activated effectors and reactivated memory T cells specific to tumor antigens [55,56]. This experimental system allowed us to track sequential changes in features of TCRα/β clonotypes involved in the primary and secondary anti-tumor immune responses. Our findings showed that CDRα of memory TCR clonotypes had stronger interactions with both MHC and docked peptides compared to CDRα of primarily activated effectors and supposedly possessed higher cross-reactivity to multiple tumor antigens. Interestingly, Dolton et al. described a T cell clone isolated from tumor-infiltrating lymphocytes (TILs) of the melanoma patient after successful TIL-therapy that expressed the TCR specific to three melanoma antigens [57]. Taking this into account, we expect that the identification of α-chain-centric TCRs of antigen-specific memory T cells with cross-reactivity to multiple antigens could be a promising strategy to improve the efficacy of ACT [55,58]. Notably, minor changes in the CDR physicochemical features of TCRβ memory clonotypes were revealed in our study, further confirming the hypothesis of the TCRα dominating role in tumor antigen recognition by memory T cells [55]. 

## 5. How Do Chain-Centric TCRs Develop?

Chain centricity could perhaps be an innate characteristic of TCR and generate stochastically during thymocyte development. The majority of studies, however, identified chain-centric receptors in TCR repertoires formed during immune responses, suggesting that antigen encounter might be crucial for the generation or selection of such receptors. This raises the question of whether chain-centric TCRs could be a feature of only those T cells that have been involved in the immune response or whether naive T cells that have not met the cognate antigen could also express such receptors.

**Figure 1:**
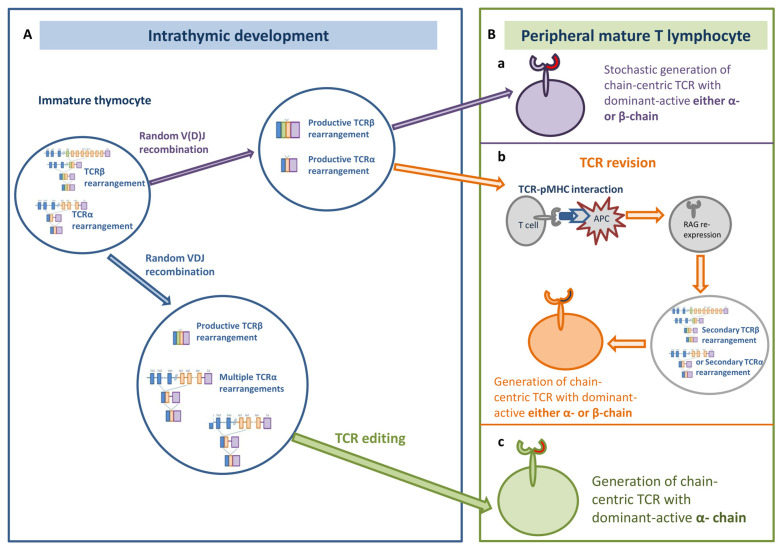
Proposed patterns of chain-centric TCR generation. During receptor formation in an immature thymocyte (**A**), either α- or β-chain-centric TCR could be generated stochastically (**a**). Alternatively, such chain-centric TCR may result from the antigen-driven TCR revision (**b**) in a mature peripheral T cell (**B**). And only chain-centric TCRs with a dominant-active α-chain could potentially be formed during TCR editing in the thymus (**c**).

In this respect, one more question remains unsolved to date: what guides the formation of a receptor with a dominant-active hemi-chain? In addition to its probable stochastic generation, we propose two potential mechanisms for chain-centric TCR genesis: TCR editing in the thymus or TCR revision in the periphery (Figure 1).

### 5.1. TCR Editing as a Mechanism of Chain-Centric TCR Formation

TCR editing, as previously mentioned (Section 4.1), aims to increase the chance of the generation of a functional α-chain capable of pairing with the rearranged β-chain and forming a selectable TCRαβ. During this process, nonfunctional (out-of-frame or nonselectable) Vα-Jα rearrangements are replaced by rearrangements on the same or the second chromosome that can potentially be functional [18,19,20]. 

Multiple TCRα gene rearrangements represent an effective mechanism for increasing the rate of potentially useful thymocytes because two-thirds of TCRα gene rearrangements are expected to be out-of-frame [47]. Indeed, TCRα gene editing has been shown to be crucial for normal thymocyte development, increasing their chances for successful positive selection [18,20,47,59,60]. Some studies have also shown that secondary TCRα recombination can be induced in the absence of positively selecting pMHC complexes, enhancing thymocyte survival [17,61,62]. Notably, non-cycling double-positive CD4+CD8+ thymocytes, already displaying TCRαβ on their surface, were found to also express RAG-1 and RAG-2 [47]. This indicates that thymocytes with a non-selectable receptor continue to rearrange TCRα loci to generate an α-chain with new specificity and replace the surface TCR by continuously making new α/β-combinations until positive selection occurs [19,47], resulting in down-regulation of RAG expression [63,64,65]. Furthermore, TCR editing was found to be involved in thymic negative selection, as secondary TCRα rearrangements allow potentially autoreactive thymocytes to survive by changing TCR specificity [16,19].

Apparently, the ability of newly generated α-chains to ensure TCR contacts with peptide-MHC complexes is the primary factor under selection. Consequently, the development of α-chain-centric TCRs may enhance the effectiveness of positive selection in the thymus. Considering the tough dependence of normal thymocyte development on the formation of proper (selectable) TCRαβ and, hence, TCR editing, this process could be proposed as a mechanism of chain-centric TCR formation. If this is the case, some naive (unprimed, antigen-unchallenged) peripheral T cells may express chain-centric TCR exclusively with a dominant-active α-chain as a result of their thymic development. Dietrich et al.’s study of the pre-immune repertoire of human immature thymocytes and peripheral mature T cells specific to the melanoma antigen melan-A in complex with the HLA-A2 allele [39] may lend some support to this hypothesis. These T cells displayed the α-chain-centric TCR resulting from selection during thymic development [39]. 

TCR editing, however, is unlikely to fully explain the phenomenon of TCR chain centricity, as receptors with dominant-active β-chains that are not subjected to TCR editing have also been described (as discussed in Section 3) (Table 1). 

### 5.2. TCR Revision as a Mechanism of Chain-Centric TCR Formation

It has been hypothesized that RAG expression is irreversibly halted in mature T cells, so the formed TCRαβ combination is conserved and cannot be changed in a given peripheral T cell. Evidence of peripheral TCR repertoire reshaping was not in alignment with the classical dogma, though. 

The process of secondary TCR gene rearrangements and the emergence of a novel antigen receptor in mature peripheral T cells is known as TCR revision [66,67,68,69,70,71]. It begins with the interaction of TCR with a peptide–MHC complex, leading to receptor internalization [72,73,74,75,76]. The loss of TCR signaling likely induces the re-expression of the recombinase machinery [67,75,77]. Many studies indicate that TCR revision indeed occurs in mature peripheral T cells and is not limited to recent thymic emigrants [66,67,69,71,77,78,79,80]. T lymphocytes with revised TCRs were found to display a diverse repertoire, respond to homeostatic signals, and recognize self and foreign peptides in the context of self-MHC molecules, indicating their advantage to the host [81]. 

TCR revision was proposed to contribute to the extrathymic expansion of the TCR repertoire [78], and we hypothesized that it could be implicated in memory cell TCR repertoire development by modifying the specificity of T cells generated during the immune response [82]. This hypothesis was supported by the evidence of an increase in memory cell responses to the cognate antigen in TCRα knockout heterozygous (TCRα^+/−^) mice with a limited ability to rearrange α-chains TCR [82]. TCR revision could also be viewed as a mechanism for the modulation of T cell functional avidity [83]. Studies of public TCRs revealed that conserved CDR3 sequences were detected in T cell populations chronically exposed to their selecting antigen, and such TCRs had similar avidities [84,85]. These findings suggest that the use of preferential CDR3 could result from TCR affinity focusing [38]. Furthermore, Miconnet et al. showed that TCRβ revision in CD8+ T cells upon viral antigen re-encounter was associated with increased T cell functional avidity [86]. In contrast to that, however, chronic HIV-1 infection, which is associated with significant changes in the clonotypic composition of specific CD8+ T cells, forced the preferential loss of high-avidity clones [87].

As TCR revision in peripheral T cells can involve either α- or β-chain gene loci and may be implicated in the changes of antigen specificity and/or functional avidity of the receptor, this process could perhaps better explain the phenomenon of TCR chain centricity. Since the revision of TCR is driven by its interactions with pMHC, this could also help explain why chain-centric receptors were mainly described for antigen-challenged T cells (Section 3). Additional in-depth investigations into the mechanisms underlying the formation of chain-centric TCRs will reveal the essential characteristics of these receptors’ genesis and enable the development of efficient methods for their identification.

## 6. Applications of Chain-Centric TCRs in Cancer Immunotherapy: Perspectives to Overcome Current Challenges

**Figure 2:**
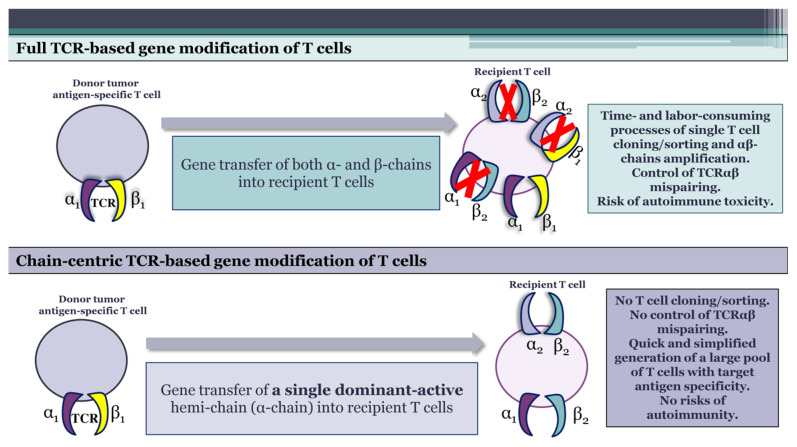
Improvement of T cell gene modification for adoptive cell therapy via the application of chain-centric TCRs. Unlike the modification of recipient T cells with full-length antigen-specific TCRs, the application of chain-centric TCRs implies T cell gene modification with a single dominant-active hemi-chain. This enables the quick and simplified generation of therapeutic T cell products with target antigen specificity, avoiding laborious T cell sorting or cloning and TCRαβ mispairing, which could pose autoimmune risks.

Far exceeding USD 350.000, ACT is currently one of the most expensive cancer treatments [88,89]. The majority of costs are incurred during the labor- and time-intensive process of developing a TCR-modified T cell product, which involves the identification of tumor-specific T cell clones, the selection of therapeutic TCRs, the T cell cloning or amplification of both α- and β-chains from single T cells, and the control of proper TCRαβ pairing in host T cells. We assume that the application of chain-centric TCRs is advantageous in many aspects and could significantly contribute to the development of more effective and safe T cell products for ACT while greatly reducing its expenses (Figure 2, Table 2).

### 6.1. Identification of Tumor-Specific TCRs

The selection of a therapeutic TCR remains one of the main challenges for ACT [1,2] and is largely limited by the low frequencies and narrow diversity of tumor-specific T cells naturally occurring in a patient’s organism [90]. Various immunotherapeutic approaches (cancer vaccines, oncolytic viruses, immune checkpoint inhibitors, etc.) could be applied to stimulate anti-tumor responses de novo, thus increasing the diversity of the tumor antigen-specific TCR repertoire [91]. Patients, however, frequently fail to respond to anti-cancer immunotherapy [91].

Therefore, different approaches have been intensively developed to facilitate the identification of tumor-specific TCRs suitable for ACT. A number of protocols were established to generate neoantigen-specific T cells in HLA-matched healthy donors, with the potential of using donor-derived tumor-specific TCRs in adoptive immunotherapy [92,93]. Up to six weeks were estimated to be required for the generation and identification of donor neoantigen-specific TCRs, the expansion of T cell clones, and their functional characterization [93]. Still, considering that, under the pressure of host anti-tumor immunity, malignant cells are often subjected to immunoediting and lose neoantigen expression, laborious and time-consuming procedures for the selection of donor-derived therapeutic TCRs could be disadvantageous. 

Large-scale bioinformatics analyses showed that TCRs specific to the same epitope share similar sequences [94,95], and groups of T cell clones with such homologue sequences (clusters) are often involved in the ongoing immune response [96]. Statistical approaches based on TCR cluster analysis have been developed (e.g., Antigen-specific Lymphocyte Identification by Clustering of Expanded sequences (ALICE)) [96], and these have allowed the identification of TCR clonotypes involved in the immune response, including anti-tumor responses, with high efficiency without longitudinal data collection [96,97]. 

Our recent work showed that the CDR3 region of the TCRα clonotypes of tumor-specific memory T cells clearly differed from the CDR3α of primarily activated effector clonotypes in its physicochemical features and was enriched with hydrophobic, strongly interacting, and bulky amino acids [55]. Hence, in combination with other bioinformatics strategies, the analysis of TCRα/β physicochemical characteristics could be a reliable approach to defining clonotypes in the patient’s bulk TCR repertoire, which are involved in anti-tumor responses. We assume that the findings of our study [55] also support the hypotheses that a naturally forming repertoire of memory T cells contains chain-centric TCRs and that α-chain-centric TCRs are likely to prevail.

Once identified, a single dominant-active hemi-chain TCR can be gene transferred into the patient’s T cells to rapidly generate a large pool of tumor antigen-specific T cells, ready to accomplish their effector functions [4,5]. This method skips cell sorting and scrupulous single-cell paired TCRαβ amplification, considerably decreasing time and labor costs for the generation of therapeutic T cell products.

### 6.2. Modulation of TCR Affinity to Target pMHC

For cancer immunotherapy, the selection of therapeutic TCRs also depends on the spectrum of antigens expressed in a given tumor. Tumor neoantigens, cancer germline antigens, and oncoviral antigens are preferentially selected as targets and considered potentially safe and efficient for TCR-T cell ACT [2,98]. Still, tumor-associated antigens are frequently more available targets that are mostly self-antigens. Therefore, only low-affinity TCRs could be found for such antigens due to the elimination of highly autoreactive T cell clones during thymic selection or via the mechanisms of peripheral tolerance [8,99].

Considering that high TCR-pMHC binding affinity/avidity is required to reach the clinical benefit of TCR-T cell ACT, many efforts are put into improving TCR affinity to chosen tumor antigens (reviewed in [58]). However, affinity enhancement of the TCR increases risks of off-tumor on-target toxicity because TCR-engineered T cells can recognize the specific pMHC expressed at low levels in non-tumor tissues. Moreover, affinity-enhanced TCRs can lose their precise specificity to a chosen pMHC and acquire cross-reactivity to self-antigens, increasing the risk of off-tumor off-target toxicity when TCR-modified T cells target normal tissues expressing different pMHC complexes [100]. 

The works of Ochi et al. [9] and Nakatsugawa et al. [10] showed that pairing the dominant-active hemi-chain with a non-dominant counter-chain can modulate TCR affinity and cross-reactivity without perturbing its antigen specificity. This feature of chain-centric receptors potentially allows the generation of therapeutic TCRs with minor off-target and cross-reactivity.

### 6.3. Control of Introduced TCRαβ Mispairing

In TCR-T cell gene modification, the precise control of correct TCRαβ pairing in host T cells is critical because the mispairing of introduced TCRα/β with an endogenous counter-chain can result in TCRs with unpredictable specificities, posing risks of autoimmune toxicity when modified T lymphocytes are infused into a patient [101,102]. Strategies to prevent TCRαβ mispairing have been extensively reviewed elsewhere [98,103,104] and include the gene silencing of endogenous TCRαβ, different modifications of TCR constant domains, the covalent linking of introduced Vα and Vβ with a polylinker, and others [98].

The introduction of a dominant-active hemi-chain of chain-centric TCR into host T lymphocytes via either electroporation, viral transduction, or CRISPR/TALEN-based techniques creates receptors with the predicted antigen specificity, the same as that of the original chain-centric receptor. Thus, the application of chain-centric TCRs could simplify T cell modification, avoiding additional gene editing to produce therapeutic T cell products.

**Figure 3:**
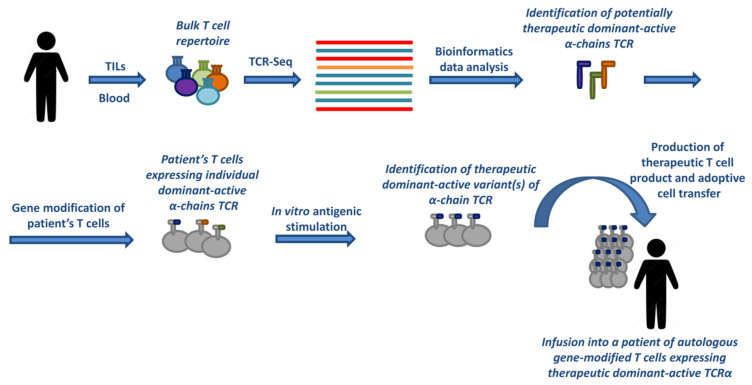
Generation of therapeutic T cell products for adoptive cancer immunotherapy based on α-chain-centric TCRs. T cells isolated from tumor-infiltrating lymphocytes (TILs) or the peripheral blood of a cancer patient are subjected to NGS sequencing in bulk to generate a TCR clonotype library. Different bioinformatics strategies are then applied to identify potentially therapeutic dominant-active α-chains TCR. Subsequently, the patient’s T cells are modified to express individual variants of these TCRα, followed by cognate antigenic stimulation in vitro and the selection of therapeutic dominant-active TCRα. Then, a therapeutic T cell product is generated via the gene modification of a patient’s T cell with the selected individual TCRα.

The schematic workflow for this strategy is presented in Figure 3. To assess the suitability of this experimental approach for clinical application and predict its possible safety concerns, we recently conducted a preclinical evaluation of the toxic effects of an experimental TCRα-modified T cell product generated via the viral transduction of mouse T cells [105]. No in vivo acute toxicity, immunotoxicity, or in vitro mutagenic or tumorigenic activity of TCRα-transduced T cells were detected in wide dose ranges, suggesting the potential safety of such T cell products [105].

Furthermore, we performed a comprehensive analysis of transgenic mice expressing the dominant-active TCRα [4,46] to deepen our understanding of the physiological and functional consequences of α-chain-centric TCR expression. Our studies showed that the specific response was profoundly enhanced in TCRα-transgenic mice due to the inborn generation of antigen-specific T cells ready to accomplish their immune functions [4,46]. Nonetheless, responses to third-party antigens were not compromised in these mice, indicating no contraction of the immune system’s reactivity because of the dominant-active TCRα expression. Furthermore, no autoimmune processes developed in these mice [46], which could be potentially attributed to the ineffective negative thymic selection of self-reactive clones generated by the random pairing of the dominant-active TCRα with endogenous β-chains. 

## 7. Conclusions

Despite the fact that chain-centric TCRs were only given this name in 2015, the asymmetric contribution of an α- and β-chain TCR to self-MHC-peptide interaction and recognition has been recognized for decades. However, little is known about the actual proportion of chain-centric TCRs in the native repertoire of mature T lymphocytes. The rate of α-chain-centric and β-chain-centric TCRs is also yet to be determined. Still, based on the developmental, structural, and functional features of the α-chain, it is plausible to propose that TCRα dominance could be a more frequent phenomenon. 

Another unresolved fundamental question is the origin of chain-centric TCR—whether it is generated during thymic development by stochastic V(D)J rearrangements or TCR editing or whether it is a result of TCR revision in the periphery. In close connection to this, a possible correlation between TCR chain-centricity and the antigen experience of a T cell expressing such a receptor is also an open question. The experimental data presented in this review support the hypothesis that chain-centric TCRs could be a feature of primed T cells and that antigen encounter might be crucial for the generation or selection of such TCRs. Comprehensive studies on the frequency of chain-centric TCRs in various functional T lymphocyte subsets (antigen-unchallenged, effector, or memory T cells) will help shed light on the genesis of such TCRs and develop efficient strategies for their identification.

Our studies confirmed that chain-centric TCRs are frequently contained in a naturally formed pool of memory T cells. Furthermore, we identified the unique physicochemical features of the CDRα of antigen-specific memory T cell clonotypes, and the results of our studies could contribute to the development of bioinformatics strategies for searching tumor antigen-specific chain-centric TCRs in bulk T cell repertoires. And although there are still many mysteries around TCR chain centricity, its applicability in T cell gene modification technologies could undoubtedly be advantageous. For cancer adoptive cell therapy, the transfer of a dominant-active α- or β-chain TCR could be a novel strategy for the generation of therapeutic T cell products with the potential to lower the current costs and improve the efficacy and safety of such treatment.

## Figures and Tables

**Table 1:** Summarizing the experimental data on chain-centric TCRs.

Experimental Setup	Antigen	Dominant-Active Hemi-Chain TCR	Reference
Mouse model	*Salmonella typhimurium*	α-chain	[5]
Mouse model	IA^b^-3K	α-chain dominates in pMHC interaction	[21]
Mouse model	p-azobenzenearsonate	α-chain	[23]
Mouse model	influenza A virus	β-chain	[24]
Mouse model (RT-1 clone)	HIV envelope glycoprotein gp160	α-chain	[25]
Mouse model (P14 clone)	lymphocytic choriomeningitis virus	α-chain	[25,26,27]
Mouse model	myelin oligodendrocyte glycoprotein	public β-chain dominates in pMHC interaction	[28]
Mouse model	K^b^-dEV8	α-chain dominates in pMHC interaction	[29]
Mouse model (D10 clone)	IA^k^-restricted	α-chain dominates in pMHC interaction	[30]
Mouse T cell hybridomas (2B4 and 2H10)	moth cytochrome *c*	β-chain dominates in pMHC interaction	[31]
TCRα transgenic mice	H-2K^b^ alloantigen	α-chain	[4]
TCRβ transgenic mice	chicken ovalbumin	β-chain	[32]
TCRβ transgenic mice	collagen type II	β-chain	[33]
TCRβ transgenic mice	autoimmune encephalomyelitis	public β-chain	[28]
Human T cell clone (TAK1 clone)	Wilms tumor antigen	β-chain	[9]
Human T cell clone (SIG35α clone)	MART-1	α-chain	[10]
Human T cell clones	HLA-DR-tt830-844 (tetanus toxin-derived peptide)	public β-chain dominates in pMHC interaction	[34]
Human CTLs *	HLA-B8/FLRGRAYGL (peptide derived from the latent antigen EBNA 3A of Epstein–Barr virus)	public α-chain dominates in pMHC interaction	[35]
Human CTLs with TCRAV12-2 gene segment	HLA-A2/ELA (peptide derived from MART-1 antigen)	α-chain	[36,37,38]
Human CTLs	HLA-A2-YVL (peptide derived from the lytic protein BRLF1 of Epstein–Barr virus)	α-chain	[38]
Human CTLs	HLA-A2-GCL (peptide derived from the lytic protein BMLF1 of Epstein–Barr virus)	α-chain	[38]
Human CTLs	HLA-B16-IE1 (peptide derived from cytomegalovirus)	α-chain	[38]
Human pre-immune repertoire (immature thymocytes and peripheral mature T cells)	HLA-A2-melan-A	α-chain	[39]

* CTL—cytotoxic T lymphocytes.

**Table 2:** Advantages of chain-centric TCRs vs. full-length TCRs for adoptive T cell therapy.

T Cell Gene Modification	
Full TCR	Chain-Centric TCR
Laborious selection of therapeutic variants	Simplified identification of potentially therapeutic variants in a bulk TCR repertoire
T cell cloning/sorting	No T cell cloning/sorting
Amplification and gene transfer of both α- and β-chain TCRs	Amplification and gene transfer of a single dominant active hemi-chain TCR
Control of mispairing with endogenous TCRαβ	No mispairing: a transferred dominant-active hemi-chain TCR combined with an endogenous counter-chain creates a TCR with target specificity
Risks of autoimmune toxicity	Minimal risks of off-target toxicity

## Data Availability

Not applicable.

## Abbreviations

TCR—T cell receptor; MHC—major histocompatibility complex; pMHC—peptide-MHC complex; ACT—adoptive cell therapy; RAG—recombination activating genes; CDR—complementarity-determining region; HLA—human leukocyte antigen; CTL—cytotoxic T lymphocyte.
